# Identification of Gene and MicroRNA Signatures for Oral Cancer Developed from Oral Leukoplakia

**DOI:** 10.1155/2015/841956

**Published:** 2015-05-03

**Authors:** Guanghui Zhu, Yuan He, Shaofang Yang, Beimin Chen, Min Zhou, Xin-Jian Xu

**Affiliations:** ^1^Department of Mathematics, Shanghai University, Shanghai 200444, China; ^2^Laboratory of Oral Biomedical Science and Translational Medicine, School of Stomatology, Tongji University, Middle Yanchang Road 399, Shanghai 200072, China; ^3^Shanghai Tenth People's Hospital, Middle Yanchang Road 30, Shanghai 200072, China

## Abstract

In clinic, oral leukoplakia (OLK) may develop into oral cancer. However, the mechanism underlying this transformation is still unclear. In this work, we present a new pipeline to identify oral cancer related genes and microRNAs (miRNAs) by integrating both gene and miRNA expression profiles. In particular, we find some network modules as well as their miRNA regulators that play important roles in the development of OLK to oral cancer. Among these network modules, 91.67% of genes and 37.5% of miRNAs have been previously reported to be related to oral cancer in literature. The promising results demonstrate the effectiveness and efficiency of our proposed approach.

## 1. Introduction

Oral leukoplakia (OLK) is one of the most common malignant disorders of the oral mucosa. There are about 2% to 3% of OLK cases that develop into oral cancers annually [1]. Therefore, the early diagnosis of the risk of OLKs developing into oral cancers can help prevent the disease process with timely and effective intervention. Unfortunately, it is still unclear how the OLKs develop into oral cancer until now. Recently, with the emergency of microarray technology that can monitor thousands of genes simultaneously, gene biomarkers are being detected for oral cancers. For example, Saintigny et al. [2] defined a 29-transcripts signature while Kondoh et al. [3] defined another 11-genes signature that can help separate oral cancers developed from OLKs from normal OLKs. Despite the good discrimination capacity of the transcript signature, few of the genes in the signature have functional relationships which make it difficult to understand the malignant transformation of oral leukoplakia.

In this work, we developed a novel pipeline to detect genes that play important roles in the development of oral cancer from a systematic perspective. Based on the protein-protein interaction (PPI) network and gene expression profiles, we detected the network modules that are associated with oral cancer development. In particular, we identified microRNAs (miRNAs) that regulate the oral cancer associated modules. Both the genes from our identified network modules and those miRNA regulators are found to be indeed related to the development of oral cancers, indicating the important roles of these modules and their miRNA regulators in the pathogenesis of oral cancer.

## 2. Materials and Methods

**Figure 1:**
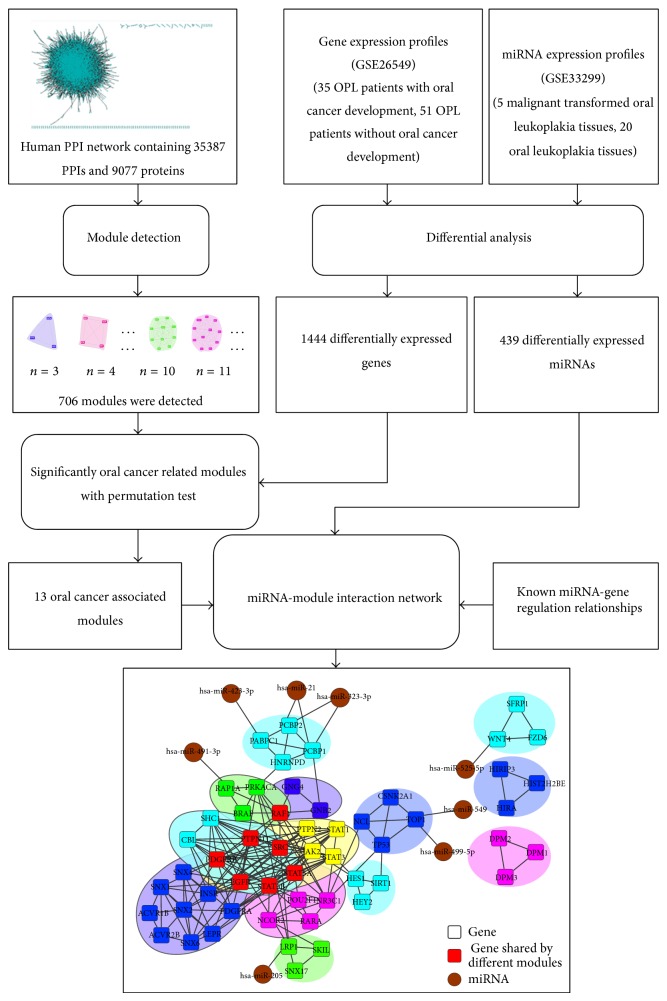
Schematic illustration of the pipeline to construct oral cancer associated network and detect key genes.

Figure 1 depicts our proposed pipeline to identify networks modules as well as their miRNA regulators that play important roles in the development of oral cancer.

### 2.1. Gene Expression

Two independent datasets (GSE33299, GSE26549) were downloaded from the NCBI Gene Expression Omnibus (GEO) [4]. The GSE33299 [5] dataset consists of miRNA expression profiles measured in three different tissues, including normal mucousal tissue (5 samples), OLK tissue (20 samples) and malignant transformed OLK tissue (5). The gene expression data were preprocessed with background subtraction and normalization using the global lowess regression model, and the missing expression values in the data were generated with Impute package in R [6]. The GSE26549 [2] dataset contains gene expression profiles measured in 35 OLK patients who developed oral cancer in follow-up time and 51 OLK patients that develop to oral cancers. All the expression data were normalized with quantile normalization and the robust multi-array average (RMA) approach. Specifically, the expression value of the gene associated with multiple probes was calculated as the average expression value of all related probes.

### 2.2. Identification of Differentially Expressed Genes and miRNAs

The miRNAs that are differentially expressed between OLKs and malignant transformed OLKs were detected by student's *t*-test with *P*-value cutoff of 0.01. As a result, 439 differentially expressed miRNAs (DEmiRs) were detected. Similarly, 1444 differentially expressed genes (DEGs) were detected by comparing their expression in OLK patients with or without oral cancer consequence with the help of student's *t*-test (*P*-value cutoff of 0.01).

### 2.3. Identification of Modules Associated with the Development of Oral Cancer

The human PPIs consist of 35387 PPIs among 9077 proteins (self-interactions excluded) were collected from the HPRD database [7]. The network modules were detected from the PPI network by utilizing CFinder [8] with default parameters. As a result, there are in total 706 modules that were identified. For each module, we defined a score *S* to measure its relevance to the development of oral cancer as follows:

(1) $$\begin{array}{c} S=\frac{\sum_{i=1}^{m}t_{i}}{m}, \end{array}$$

where *m* denotes the number of genes in the module, *t*_*i*_ denotes the *t*-score of gene *i* obtained with student's *t*-test analysis on the gene expression data between OLKs with or without oral cancer. Moreover, to control the false discovery rate, the *P*-value for each module was defined as the probability that this module was observed by chance. In particular, the same number of genes as that in the module was randomly picked up and the score for this gene set was calculated as described in (1). This procedure was repeated for 10000 times, and the frequency of observing a score larger than *S* was defined as the *P*-value for the corresponding module. Consequently, 13 modules were selected with *P*-value threshold of 0.01, and these modules were regarded as modules associated with oral cancer (MAOCs) and play important roles in the development of oral cancer.

### 2.4. Network Analysis

In complex networks, compared with average nodes, the hub nodes that have larger degree and link to more nodes generally play more important roles in the system. Therefore, we detected the hub genes from the 13 MAOCs. In the detail, we first defined the scaled connectivity *K*_*i*_ for the *i*th gene as follows:

(2) $$\begin{array}{c} K_{i}=\frac{k_{i}}{k_{\max}}, \end{array}$$

where the *k*_*i*_ is the degree of gene *i*, *k*_max⁡_ denotes the maximum degree of the genes in the MAOCs. Subsequently, the genes that have *K*_*i*_ larger than 0.9 were regarded as hub genes hereinafter.

Except for the hub genes, there are some genes that link distinct modules, which were also assumed to be important since they bridge the signal flows between distinct modules. In particular, we grouped these genes into two categories: (1) genes located in one module and interact with genes from other modules; (2) genes outside of MAOCs but interact with genes from other modules. Especially, for each of the above identified genes, the connecting score CS_*i*_ for the *i*th gene was defined as the number of MAOCs that this gene links to.

### 2.5. Regulations between miRNAs and Genes

Target genes of miRNAs were predicted through utilizing tools including PicTar [9], miRanda [10], MicroT [11] and TargetScan [12]. Target genes of each miRNA were kept only if they were predicted by at least two tools. Target genes of each miRNA were extended with experimentally determined miRNA target genes deposited in Tarbase [13]. A set of 23336 miRNA-gene interactions involving 548 miRNAs and 6797 genes was obtained.

### 2.6. Identification of miRNAs Regulating MAOCs

Based on miRNA-gene regulations, given one miRNA, a module will be regarded to be regulated by this miRNA if its target genes are significantly enriched in this module, where the enrichment analysis was conducted with Fisher exact test with *P*-value cutoff of 0.01. As a result, for each DEmiR, we identified the modules that can be regulated by this miRNA.

## 3. Results

### 3.1. Network Modules Associated with Oral Cancer

**Figure 2:**
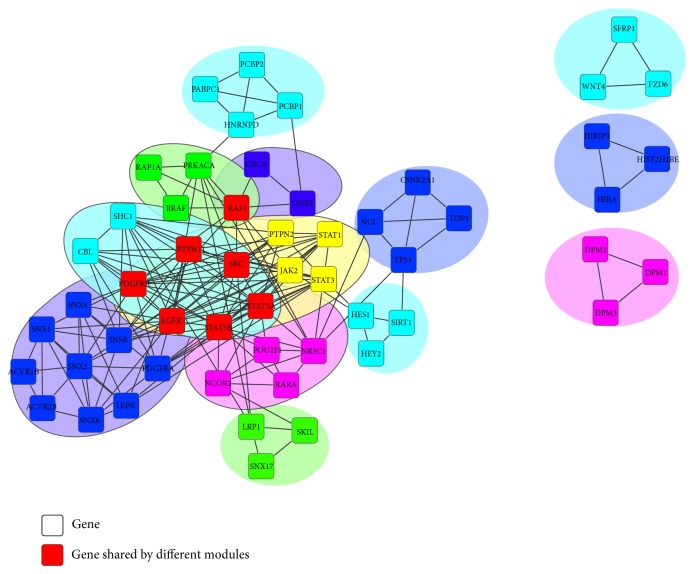
The protein-protein interaction network consists of proteins as well as their interactions from the 13 modules, where distinct modules were marked in different colors and the genes in red denote those occurring in more than two modules.

In the 13 MAOCs we identified, there are in total 54 genes involved, among which 38.89% are DEGs (more details see Section 2).  Figure 2 shows the PPI network consists of proteins involved in the 13 modules, where the interactions between proteins were obtained from the HPRD database, where distinct modules were marked with different colors while those genes occurring in more than two modules were marked in red.  Table 1 summarizes the genes from the 13 modules. Furthermore, the functional enrichment analysis was performed for these genes with the Database for Annotation, Visualization and Integrated Discovery (DAVID) service [14, 15]. From the results (supplementary Table 1 in Supplementary Material available online at http://dx.doi.org/10.1155/2014/841956) we can see that the well known processes associated with cancer development, such as cell proliferation, apoptosis, cycle, migration and differentiation, are significantly in the module genes. Moreover, the epidermal growth factor receptor signaling pathway that has been previously reported to be related to squamous cell carcinoma (SCC) [16] is significantly enriched in the module genes. In addition, the pathways in cancer and Wnt signaling pathway, which are known to be related to cancer, were also found to be significantly enriched [17]. The functional enrichment analysis results indicate that our identified modules are related to the development of oral cancer.

Since there are some genes that have already been known to be related to oral cancer, to validate the associations between our identified modules and oral cancer, we obtained a gene list of 2990 genes from the GeneCards [18] by querying “oral cancer”. Among the 54 genes in the 13 modules, 30 (55.56%) genes can be found in the 2990 genes (Table 2). That is, these 30 genes have already been annotated to be related to oral cancer. In particular, 15 of the 30 genes (50%) were found to be ranked in the top 32% of the gene list according to the relevance scores provided by GeneCards, where the relevance score was calculated by Lucene scoring to determine how relevant a given document is to a query. Considering that the SCC accounts for the majority of oral cancer cases, a list of genes was retrieved from GeneCards by querying “squamous carcinoma”. As a result, 33 (61.11%) of our module genes can be found in the 2648 genes annotated to be related to SCC, while 24 (72.73%) of the these genes were ranked in the top 54% of the gene list according to the relevance scores provided by GeneCards (supplementary Table 2). The overlap between our identified module genes and those known oral cancer genes indicates that our identified modules are indeed related to the development of oral cancer.

### 3.2. Topological Important Genes in the MAOCs

In general, the hub nodes and those linking modules play more important roles in the system underlying the network. In our identified MAOCs, we picked up 4 hub genes, 5 intra-MAOC genes and 5 inter-MAOC genes, where the intra-MAOC genes are those inside one module and have interactions with genes belonging to other modules while the inter-MAOC genes are those outside of MAOCs but have interactions with genes belonging to MAOCs.  Table 3 lists these topological important genes. Note that it is possible that some genes are both hub genes and intra-MAOC genes, for example, PDGFRB gene.

**Figure 3:**
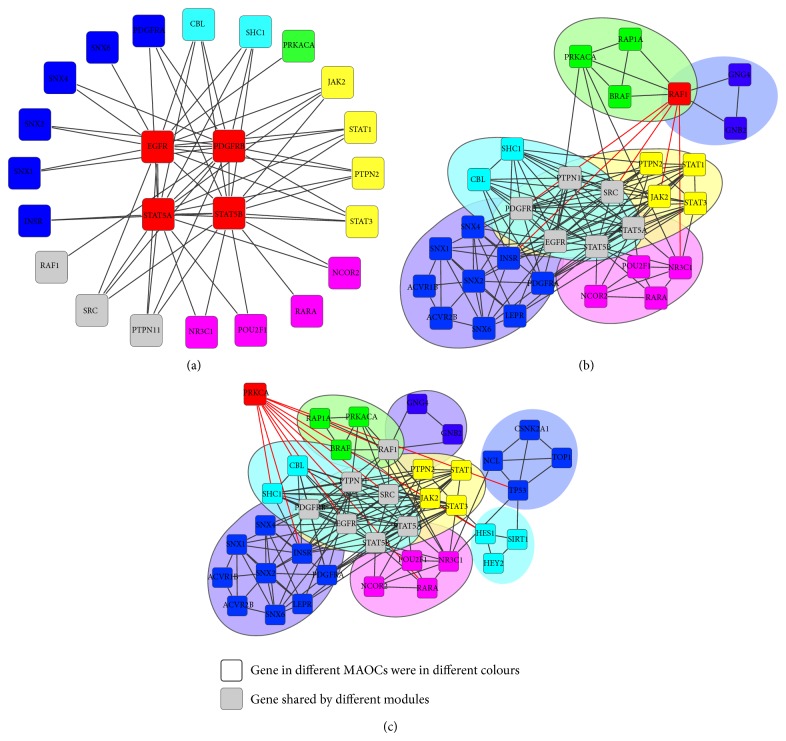
As representative for the three kinds of genes which were detected as key genes associated with oral cancer development, (a) 4 hub genes, (b) RAF1 as an intra-MAOC gene had 5 interactions between modules getting an connecting score of 8, and (c) PRKCA as an inter-MAOC gene connected 8 modules getting an connecting score of 8 were shown in this figure.

(1) The hub genes we identified have more interactions than other genes, that is, higher degree, and therefore have more important roles. Among the 4 hub genes, the three genes EGFR, PDGFRB, STAT5A have already been annotated to be related to oral cancer according to GeneCards (see Table 2). In the MAOCs, the gene STAT5B has high degree of 17. Despite this gene has not been annotated to be related to oral cancer in GeneCards, both STAT5A and STAT5B belong to the STAT5 family the aberrant activity of which has been found to be related to various cancers [19]. Especially for SCC, blockade of STAT5B in a xenograft model in head and neck squamous cell carcinoma (HNSCC) resulted in tumor growth inhibition [20], and the constitutive activation of STAT5A was one of the early events in tobacco mediated-oral squamous cell carcinoma (OSCC) in the eastern Indian population [21]. Among the other two hub genes both with high degree of 16, EGFR has been among the most important prognostic factors for HNSCC [16], while the kinase PDGFRB was found to be up regulated in tumor indicating the effectiveness of tyrosine and serine-threonine kinase inhibitors in the treatment of HNSCCs [22].

(2) Intra-MAOC genes interact with genes in distinct MAOCs which effected oral cancer development in the common pathological system. The 5 intra-MAOC genes with highest connecting scores were identified as key genes, all of which are found to be related to oral cancer according to GeneCards (see Table 2). For example, the gene RAF1 (Figure 3) has a medium degree of 10 and the highest connecting score of 8, implying that RAF1 is important for oral cancer development although it is not a hub gene. In fact, RAF1/Rok-*α* interaction has been found to play a critical role in the pathogenesis of SCCs [23]. Except for the hub genes PDGFRB and EGFR, SRC has also been previously reported to be related to SCC with increased expression in HNSCC [24].

(3) Inter-MAOC genes are those outside of MAOCs and interact with genes in more than 2 distinct MAOCs. Similarly, the top 5 inter-MAOC genes with the highest connecting scores were identified as key genes. For example, the PRKCA gene with the top connecting score of 8 (Figure 3) interacts with 6 genes (INSR, EGFR, RARA, SRC, RAF1, TP53) from MAOCs, where these 6 genes have been reported to be oral cancer related genes according to GeneCards (see Table 2). Therefore, it is reasonable to conclude that PRKCA plays important roles in the development of oral cancer. Actually, PRKCA was found to be highly expressed in HNSCC [25]. Moreover, it was found that the phosphorylation of MAPK1/MAPK3 was correlated with tumor growth in OSCC [26], the methylation of ESR1 promoter was associated with squamous cell cervical cancer [27], and the activation of FYN kinase was related to OSCC [28].

From above results, we can see that most of our identified key genes (11/12) have been reported previously to be associated with oral cancer, which demonstrate that our identified network modules are indeed related to the pathogenesis of oral cancer.

### 3.3. miRNAs Regulators of Network Modules

**Figure 4:**
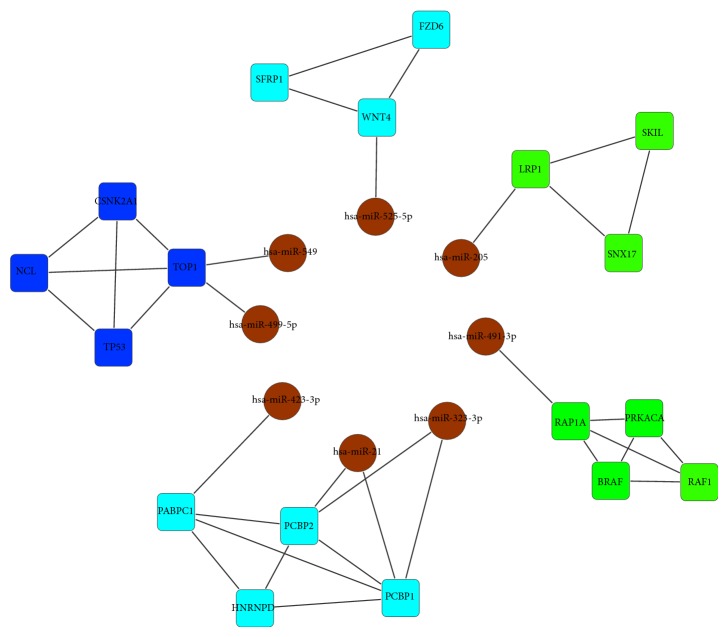
The miRNA-module interaction network, where the nodes in circle are miRNAs and the rest are genes and distinct modules were marked in different color.

Among our identified 439 differentially expressed miRNAs (DEmiRs), 8 DEmiRs were found to regulate 5 modules as shown in Figure 4. By investigating the expression profiles of these DEmiRs as well as those of their target genes, we noticed that the expression of 4 miRNAs is negatively correlated with that of their target genes (Table 4). For example, the expression of hsa-miR-491-3p is down-regulated in oral cancers samples while the expression of its corresponding target gene RAP1A is up-regulated in oral cancers, which is consistent with the general miRNA regulation principle that miRNAs repress the expression of their target genes. In literature, RAP1A has been reported as a critical mediator of HNSCC [29]. Moreover, in module 13, RAP1A interacts with BRAF and RAF1 that have also been reported to be associated with the progression of SCC [23, 30–32]. The evidence from literature makes it clear that module 13 is indeed related to the oral cancer, and it is reasonable that the regulator, that is, hsa-miR-491-3p, of this module also play important roles in the development of oral cancer.

Another example is hsa-miR-21 that regulates the module 12 by directly regulating its target genes PCBP1 and PCBP2. In the oral cancers, hsa-miR-21 was found to be up-regulated while its target genes PCBP1 and PCBP2 were found to be down-regulated. The two genes PCBP1 and PCBP2 have been reported to be related to SCC [33, 34] in literature. In module 12, the four member genes interact with each other and form a clique structure. Among the 4 genes, the knockdown of HNRNPD has been found to reduce the hTERT promoter activity in OSCC while PABPC was shown as critical in hTERT regulation by human papillomavirus 16 E6 which had been associated with HNSCC [35–37]. It is obvious that module 12 plays important roles in SCC. Except for miR-21, another two miRNAs miR-323-3p and miR-423-3p were also found to regulate module 12. As the regulators of module 12, and we believe its miRNA regulators should also be related to SCC. In fact, the deregulation of miR-21 has found to be related to HNSCC in literature [38], and the higher expression of miR-21 was associated with shortened survival time in squamous cell lung carcinoma [39].

Except for the above two miRNAs, another two hsa-mir-499 and miR-205 have also been reported to play important roles in OSCC in literature. It was found that hsa-mir-499 was associated with the reduced risk of HNSCC [40] and miR-205 can be used as a biomarker in discriminating SCC from adenocarcinoma and small cell lung carcinoma with high accuracy [41]. The evidence supports from literature demonstrate that the miRNA regulators of our identified modules are indeed related to the development of oral cancer.

## 4. Conclusions

The oral leukoplakia frequently develops into oral cancers, however, the mechanism underlying which is still unclear. In this work, with the assumption that the oral cancers were caused due to the dysfunction of multiple functionally related genes [42], we proposed a novel pipeline to identify gene modules that play important roles in the development of oral cancers. The genes in our identified modules were found to be indeed related to oral cancer and the miRNA regulators of these modules were also reported in literature to be related to the cancer, indicating the effectiveness of our proposed approach. In particular, we identified some key genes that play crucial roles in the malignant transformation of oral leukoplakia to oral cancer, including 4 hub genes (STAT5B, EGFR, PDGFRB, STAT5A), 5 intra-MAOC genes (RAF1, PDGFRB, SRC, PRKACA, EGFR), 5 inter-MAOC genes (PRKCA, MAPK1, MAPK3, ESR1, FYN) and 8 miRNAs (hsa-miR-499-5p, hsa-miR-549, hsa-miR-205, hsa-miR-525-5p, hsa-miR-21, hsa-miR-323-3p, hsa-miR-423-3p, hsa-miR-491-3p). These findings may provide new clues for future research.

This work also supplies a feasible work frame to detect key genes and miRNAs in development of other special type of caner based on gene and miRNA expression profiles. However the process of identification is largely based on the acknowledged PPI network that is not yet complete, so our method is supposed to achieve more realistic detection while the PPI network is more complete and reliable. On the other hand, considering that the process of identification is depend on the algorithm to detect the modules in PPI network and the parameters set to the algorithm and both the algorithm and the special parameters are not designed based on evolving nature of relations between molecules in cancer development but mathematical logic, selecting module detecting algorithm that can exploit the significance in biological process is supposed to improve the identification capacity of our method.

## Supplementary Material

supplementary Table 1: Results of the functional enrichment analysis for the genes from each one of the 13 modules identified by us associated with oral cancer development.
supplementary Table 2: 33 genes in the 13 modules identified by us associated with oral cancer development and their relevance score representing correlation degree with SCC provided by GeneCards.

## Figures and Tables

**Table 1:** The modules we identified to be associated with oral cancer, and the enriched functions and pathways of the proteins from these modules.

	Genes in module (DEGs were in bold)	Top 10 enriched biological processes	Top 10 enriched KEGG pathways
Module 1	DPM3 DPM1 **DPM2**	GO: 0032870 cellular response to hormone stimulus	hsa05220: Chronic myeloid leukemia
Module 2	**HIRA HIST2H2BE HIRIP3**		
Module 3	EGFR PDGFRB PTPN11 SHC1 **SRC STAT5A STAT5B** CBL	GO: 0007169 transmembrane receptor protein tyrosine kinase signaling pathway	hsa05200: Pathways in cancer
Module 4	CSNK2A1 NCL **TOP1 TP53**		
Module 5	NR3C1 **POU2F1** RARA **STAT5A STAT5B** NCOR2	GO: 0009725 response to hormone stimulus	hsa04062: Chemokine signaling pathway
Module 6	**LRP1** SKIL SNX17	GO: 0007167 enzyme linked receptor protein signaling pathway	hsa05210: Colorectal cancer
Module 7	GNB2 GNG4 **RAF1**		
Module 8	EGFR INSR LEPR **PDGFRA** PDGFRB SNX6 **SNX1** SNX2 SNX4 ACVR1B ACVR2B	GO: 0009719 response to endogenous stimulus	hsa04012: ErbB signaling pathway
Module 9	EGFR JAK2 PDGFRB PTPN2 PTPN11 **SRC** STAT1 STAT3 **STAT5A STAT5B**	GO: 0010033 response to organic substance	hsa05214: Glioma
Module 10	**SIRT1** HEY2 **HES1**	GO: 0007166 cell surface receptor linked signal transduction	hsa05212: Pancreatic cancer
Module 11	WNT4 SFRP1 **FZD6**		
Module 12	PABPC1 **HNRNPD PCBP1 PCBP2**	GO: 0006468 protein amino acid phosphorylation	hsa05221: Acute myeloid leukemia
Module 13	PRKACA **RAF1 RAP1A** BRAF		
		GO: 0018108 peptidyl-tyrosine phosphorylation	hsa04630: Jak-STAT signaling pathway
		GO: 0018212 peptidyl-tyrosine modification	hsa05218: Melanoma

**Table 2:** The 30 genes that have been annotated to be related to oral cancer and their relevance scores according to GeneCards.

Gene symbol	Relevance score
TP53	10.19
EGFR	10.17
RARA	9.54
SRC	9.48
BRAF	9.37
TOP1	9.37
STAT3	9.32
SFRP1	9.26
RAF1	9.18
STAT1	9.1
LEPR	9.08
STAT5A	8.89
PCBP2	8.43
HNRNPD	8.32
PDGFRB	0.18
PDGFRA	−0.21
NCL	−0.29
PRKACA	−0.39
NR3C1	−0.44
CSNK2A1	−0.52
FZD6	−0.59
INSR	−0.59
PTPN11	−0.64
JAK2	−0.77
WNT4	−0.87
RAP1A	−0.95
SNX1	−1.01
PTPN2	−1.13
PABPC1	−1.16
POU2F1	−1.25

**Table 3:** The details of topological important genes, including hub genes, intra-MAOC genes and inter-MAOC genes.

Hub gene	Degree	Intra-MAOC gene	Connecting score	Inter-MAOC gene	Connecting scores
STAT5B	17	RAF1	8	PRKCA	8
EGFR	16	PDGFRB	7	MAPK1	7
PDGFRB	16	SRC	6	MAPK3	7
STAT5A	16	PRKACA	6	ESR1	6
		EGFR	4	FYN	6

**Table 4:** The miRNA regulators of modules and their target genes.

miRNA (in bold if the expression of miRNA and that of its target gene is negatively correlated)	miRNA Expression change direction	miRNA target gene	Gene Expression change direction
hsa-miR-499-5p	Up	TOP1	up
hsa-miR-549	Up	TOP1	up
**hsa-miR-205**	Up	LRP1	down
hsa-miR-525-5p	down	WNT4	down
**hsa-miR-21**	Up	PCBP1 PCBP2	down down
hsa-miR-323-3p	down	PCBP1 PCBP2	down down
**hsa-miR-423-3p**	down	PABPC1	up
**hsa-miR-491-3p**	Up	RAP1A	down
